# Correction: Microglial inflammation in the parkinsonian substantia nigra: relationship to alpha-synuclein deposition

**DOI:** 10.1186/1742-2094-3-9

**Published:** 2006-04-06

**Authors:** Emilie Croisier, Linda B Moran, David T Dexter, Ronald KB Pearce, Manuel B Graeber

**Affiliations:** 1 Department of Neuropathology, Division of Neuroscience and Mental Health, Imperial College London, and Hammersmith Hospitals Trust, London, UK; 2 Department of Cellular and Molecular Neuroscience, Division of Neuroscience and Mental Health, Imperial College London, London, UK

Upon re-examination of all dates of birth and death of cases described in this paper [1], the ages at death of the following cases presented in Table 1 need to be corrected as follows:

**Table 1:** 

Case	Reported age at death	Actual age at death
3	71	70
6	83	82
7	73	72
8	77	76
9	75	76
12	75	74
13	83	82
14	77	76
16	83	84

All other clinical information is accurate, and this does not affect the findings of the paper.
